# Mitochondrial Respiration in *KRAS* and *BRAF* Mutated Colorectal Tumors and Polyps

**DOI:** 10.3390/cancers12040815

**Published:** 2020-03-28

**Authors:** Egle Rebane-Klemm, Laura Truu, Leenu Reinsalu, Marju Puurand, Igor Shevchuk, Vladimir Chekulayev, Natalja Timohhina, Kersti Tepp, Jelena Bogovskaja, Vladimir Afanasjev, Külliki Suurmaa, Vahur Valvere, Tuuli Kaambre

**Affiliations:** 1 Laboratory of Chemical Biology, National Institute of Chemical Physics and Biophysics, Akadeemia tee 23, 12618 Tallinn, Estonia; laura.truu@gmail.com (L.T.); leenu.reinsalu@gmail.com (L.R.); marju.puurand@kbfi.ee (M.P.); igor@chemnet.ee (I.S.); vladimir@chemnet.ee (V.C.); natalja.timohhina@kbfi.ee (N.T.); kersti.tepp@kbfi.ee (K.T.); tuuli.kaambre@kbfi.ee (T.K.); 2 Department of Chemistry and Biotechnology, School of Science, Tallinn University of Technology, Ehitajate tee 5, 12618 Tallinn, Estonia; 3 Clinic of Diagnostics at the North Estonia Medical Centre, J. Sütiste tee 19, 13419 Tallinn, Estonia; jelena.bogovskaja@regionaalhaigla.ee; 4 Clinic of Surgery at the North Estonia Medical Centre, J. Sütiste tee 19, 13419 Tallinn, Estonia; Vladimir.Afanasjev2@regionaalhaigla.ee; 5 Department of Gastroenterology, the West Tallinn Central Hospital, Paldiski mnt 68, 10617 Tallinn, Estonia; kulliki.suurmaa@keskhaigla.ee; 6 Oncology and Haematology Clinic at the North Estonia Medical Centre, J. Sütiste tee 19, 13419 Tallinn, Estonia; vahur.valvere@regionaalhaigla.ee

**Keywords:** energy metabolism, colorectal cancer, colorectal polyps, mitochondria, oxidative phosphorylation, *KRAS*, *BRAF*

## Abstract

This study aimed to characterize the ATP-synthesis by oxidative phosphorylation in colorectal cancer (CRC) and premalignant colon polyps in relation to molecular biomarkers KRAS and BRAF. This prospective study included 48 patients. Resected colorectal polyps and postoperative CRC tissue with adjacent normal tissue (control) were collected. Patients with polyps and CRC were divided into three molecular groups: *KRAS* mutated, *BRAF* mutated and *KRAS/BRAF* wild-type. Mitochondrial respiration in permeabilized tissue samples was observed using high resolution respirometry. ADP-activated respiration rate (V_max_) and an apparent affinity of mitochondria to ADP, which is related to mitochondrial outer membrane (MOM) permeability, were determined. Clear differences were present between molecular groups. *KRAS* mutated CRC group had lower V_max_ values compared to wild-type; however, the V_max_ value was higher than in the control group, while MOM permeability did not change. This suggests that *KRAS* mutation status might be involved in acquiring oxidative phenotype. *KRAS* mutated polyps had higher V_max_ values and elevated MOM permeability as compared to the control. *BRAF* mutated CRC and polyps had reduced respiration and altered MOM permeability, indicating a glycolytic phenotype. To conclude, prognostic biomarkers KRAS and BRAF are likely related to the metabolic phenotype in CRC and polyps. Assessment of the tumor mitochondrial ATP synthesis could be a potential component of patient risk stratification.

## 1. Introduction

Colorectal cancer (CRC) is the leading cause of premature cancer death worldwide, prompting the urgent need to develop more effective treatment strategies. CRC is a heterogeneous disease and presents distinct subtypes with different molecular and pathological features. The majority of sporadic CRC typically develops progressively from premalignant precursor lesions, known as polyps, to malignant tumors. Most colorectal polyps are harmless, but some can develop (by not fully understood mechanisms) into malignant invasive adenocarcinomas. According to modern concepts, CRC is triggered by various molecular events in several proto-oncogenes (such as the *PIK3CA*, *p53*, *KRAS*, *BRAF* and *c-MYC* genes) and tumor suppressor genes (such as the APC, PTEN, SMAD4 genes) [1,2,3]. The malignant transformation of cells, including colon epithelium, is accompanied by strong alterations (reprogramming) of metabolic pathways involved in energy production and biosynthesis that promote tumor growth and metastasis [4,5,6]. A better understanding of the pathogenesis of CRC, the metabolic heterogeneity of emerging polyps and potential drivers is very important to develop new prognostic markers and successful agents for the prevention and treatment of this disease.

Transcriptome-based classification has been used in CRC as it can better describe the behavior of the tumors. The international CRC Subtyping Consortium classifies CRC into four consensus molecular subtypes (CMSs), each with distinct features: CMS1 (hypermutated, microsatellite instability (MSI), *BRAF* mutation, and immune infiltration and activation); CMS2 (epithelial, WNT and MYC signaling pathway activation); CMS3 (metabolic dysregulation, *KRAS* mutations); and CMS4 (transforming growth factor beta activation, stromal invasion, TGFβ activation, and angiogenesis) [7]. Although transcriptome profiles are not associated with specific mutations, the frequency of *KRAS* mutation varies among the CRC subtypes (23% in CMS1, 28% in CMS2, 68% in CMS3, and 38% in CMS4), these data suggest mutations may drive distinct programs of metabolism gene expression [7]. Mutations in *KRAS* or *BRAF* genes appear to play an important role in the regulation of metabolic reprogramming in multiple cancers, including CRC [8,9,10,11]. In this study, two established and common prognostic biomarkers in CRC were investigated: *KRAS* and *BRAF* mutation status. Mutation in *BRAF* codon 600 of exon 15 (V600E) is associated with unfavorable prognosis [12]. Activating *KRAS* mutations in codon 12 and 13 of exon 2, which is common in CRC (30–50% of tumors), are associated with poorer survival and response to chemotherapeutics [13,14]. Our study aims to contribute to understanding how prognostic biomarkers KRAS and BRAF are correlating to cellular metabolic phenotypes in the course of CRC carcinogenesis.

The metabolism of cancer cells is specially adapted to meet their needs to survive and proliferate in both well oxygenated and hypoxic microenvironments. To date, transcriptomics and metabolomics studies have shown the coexistence of three distinct cellular metabolic phenotypes that exist in cancer cells, which are characterized by the following predominant states: glycolytic (aerobic glycolysis, so called Warburg phenotype [15]), oxidative (energy production relying mainly on oxidative phosphorylation, OXPHOS), and hybrid (both OXPHOS and glycolysis can be active simultaneously). Normal cells exhibit only glycolytic and oxidative states [16,17,18]. Premalignant polyps and arising adenocarcinomas are still regarded as highly glycolytic tumors of the Warburg phenotype [19,20,21]. Previous studies indicate that although polyps have higher inclination to aerobic glycolysis, the metastatic carcinomas maintain high rates of O_2_ consumption (much more than adjacent normal tissues) and exhibit obvious signs of stimulated mitochondrial biogenesis [6,22,23,24]. In this regard, we assume that upon malignant transformation, there is a selection of specific cell clones that have stimulated mitochondrial biogenesis and, as a result, have elevated aggressiveness. Among patients with CRC, a high level of mitochondrial respiration of tumor samples have been found to be associated with reduced survival [25].

As part of cancer bioenergetic studies, analysis of OXPHOS with high-resolution respirometry can be applied to study the mechanisms of this key element in cellular bioenergetics. Investigating the dependency of adenosine diphosphate (ADP)-dependent respiration rate on ADP concentration in tissue samples can provide two fundamental characteristics for OXPHOS: a maximal ADP-activated respiration rate (V_max_), and an apparent affinity of mitochondria for exogenous ADP expressed as apparent Michaelis–Menten constant Km (K_m_(ADP)). Our previous experiments showed that the V_max_ value for CRC cells is significantly higher than in cells in healthy colorectal control tissue showing more active ATP-synthesis by OXPHOS. This finding corresponds well with differences in the content of mitochondria in these cells (the number of mitochondria in CRC is almost two times higher than in healthy tissue) [6,25]. The changes in K_m_(ADP) show changes in tissue-specific intracellular complexity in terms of energy transport and regulation of mitochondrial outer membrane (MOM) permeability. For the operation of OXPHOS, the flux of respiratory substrates, ATP, ADP and Pi through MOM is regulated by the voltage-dependent anion channel (VDAC) permeability control. In the closed state, VDAC is impermeable to adenine nucleotides [26,27]. Several studies have shown that during carcinogenesis the VDAC permeability for ADP is altered [22,28,29,30]. The cell-specific differences in K_m_(ADP) are likely due to specific structural and functional organization of energy metabolism. For example, cells with a low K_m_(ADP) value (~10 µM) like glycolytic muscle, possess less structural and functional obstacles for movement ADP/ATP though MOM as compared to the oxidative muscles (~300 µM) [31]. Known K_m_(ADP) values for CRC measured for tumor tissue are about 100 µM [22,25], implying existence of some restrictions for ADP passing VDAC. The sensitivity of the mitochondrial respiration for exogenous ADP in cell cultures is very high (low K_m_(ADP) values) and is similar to isolated mitochondria [25,28,32,33,34], which suggests the need to investigate cancer energy metabolism directly in fresh clinical material. To our knowledge, there is no data on the rate of OXPHOS and its regulation in colon polyps. Assessment of OXPHOS status of this pathology enhances our understanding of colon carcinogenesis.

Thus, the main goal of our study was to characterize the functional activity of mitochondrial OXPHOS among premalignant polyps and CRC, taking into account their *KRAS* and *BRAF* mutation status. To date, it has been shown that *KRAS* and *BRAF* mutations increase the glycolytic capacity of tumor cells and their glutaminolysis [8,35]. In our work, the function of the OXPHOS system was analyzed by means of high-resolution respirometry using freshly prepared postoperative tissue samples.

## 2. Results and Discussion

Cancer metabolism profoundly differs from normal cellular metabolism, and interrelated connections between cancer mitochondrial respiration and oncogenic driver genes like *KRAS* and *BRAF* are relatively unexplored. Somatic mutations involving the GTP-ase RAS protein family and its downstream serine/threonine-protein kinase BRAF lead to loss of cell cycle regulation at key checkpoints and are the main driver mutations for colorectal carcinogenesis [36]. *KRAS* mutations are detected in approximately 40% of all CRC patients, suggesting the importance of *KRAS* in tumor development [37]. The *KRAS* mutation is an early event in CRC and most *KRAS* mutations are located in codons 12 and 13. However, at least 5–10% of CRCs are believed to initiate via acquiring activating mutations in the *BRAF* oncogene [38]. Mutations of *KRAS* and *BRAF* are usually mutually exclusive. Although the existence of intertumoral heterogeneity in CRC is well established and illustrated by molecular subtyping [7], pure genome or transcriptome data are not sufficient to describe the final in situ modifications and the final outcomes of pathways or cellular processes [25]. The purpose of this study was to determine the activity of ATP production by OXPHOS in human tissues during the development of CRC from normal colon tissue to polyps and cancer, depending on the status of *BRAF* and *KRAS* mutations.

**Figure 1:**
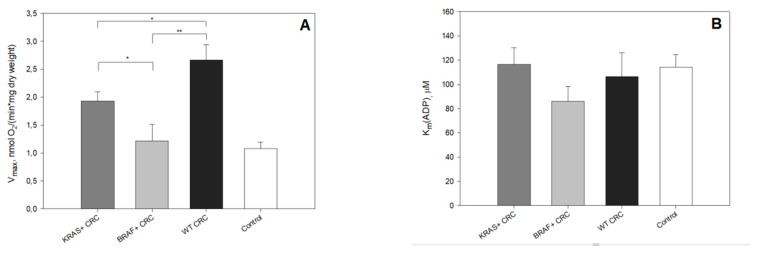
Regulation of mitochondrial respiration in *KRAS*+, *BRAF*+ and wild-type tumors and control. (**A**) Comparative analysis of maximal ADP-activated respiratory rate (V_max_) and (**B**) the apparent Michaelis–Menten constant (K_m_(ADP)) values for ADP. *KRAS*+: *KRAS* mutated; *BRAF*+: *BRAF* mutated; WT: wild type; CRC: colorectal cancer; Control: control tissue. * *p* < 0.05; ** *p* < 0.01.

To characterize ATP-synthesis by OXPHOS during CRC carcinogenesis we used high resolution respirometry to measure the rate of maximal ADP-activated respiration (V_max_). We also used apparent K_m_ values for exogenously added ADP (K_m_(ADP)) using permeabilized postoperative tissue (CRC, colon polyps and normal colon tissue). Our previous studies showed that OXPHOS can be a significant supplier of ATP in CRC because its V_max_ values (corresponding to the number of mitochondria) were almost two times higher than in surrounding normal tissues [6,39,40]. Among all the studied groups, the wild-type tumor showed the highest V_max_, while these values measured for *BRAF* or *KRAS* mutated tumors were significantly lower (Figure 1A, Tables S1 and S2). This reveals involvement of oncogenic *KRAS* and *BRAF* in metabolic reprogramming of colon mucosa and confirms their role in shifting CRC metabolism to a more glycolytic type. Furthermore, in contrast to the results from an in vitro study conducted by Yun et al.—done with CRC cell cultures where oxygen consumption in cells with mutant *KRAS* or *BRAF* alleles was similar to that in cells with wild type alleles of these genes [41]—we saw a difference in V_max_ values between *BRAF* mutated and *KRAS* mutated tumors (Figure 1A, Tables S1 and S2). Interestingly, the V_max_ of *BRAF* mutated tumors was similar to that in control tissues. These results suggest a distinct role of mutated *KRAS* and *BRAF* in affecting mitochondrial biogenesis and likely tissue differentiation as well.

In colorectal polyps, the V_max_ pattern largely followed that of the respective tumors. The respiration rates in polyps in *KRAS* mutated and wild-type molecular groups showed remarkably higher V_max_ values than the control tissue (V_max_ values 2.19 ± 0.19 and 1.95 ± 0.28 for *KRAS* mutated and wild-type group, respectively, *p* < 0.001 and *p* = 0.004 as compared to the control group (Tables S1 and S2). Polyps that had acquired the *BRAF* mutation showed a tendency to have lower OXPHOS rates (V_max_ 1.41 ± 0.27) than in mutated *KRAS* and wild-type groups. Similar to the *BRAF* tumor group, polyps with mutated *BRAF* did not show a difference with the control tissue (Figure 1, Tables S1 and S2). This suggests that alterations in mitochondrial biogenesis is a very early event and already happens in the pre-malignant stage.

Maintaining high functional activity of OXPHOS may be necessary because cancer cells with a very low respiration rate cannot form tumors [42]. At the same time, a certain reduction in respiration may be useful for the functioning of signaling molecules, the synthesis of anabolic precursors and other typical aspects of cancer phenotypes [43]. Thus, functional OXPHOS is important in both proliferating and non-proliferating cells, but each situation will emphasize its unique functional aspects [42]. It has been shown that the metabolic profile of cancer cells in culture can have significant variations as a consequence of the culture conditions [25]. In general, cells growing in a glucose-free medium display relatively high rates of oxygen consumption, whereas cultivation in a high-glucose medium results in hyperglycolytic cells together with declined respiratory flux [44,45,46,47,48]. Therefore, for the study of OXPHOS in human tumors, the use of postoperative tissue material is likely to be a more suitable approach.

To investigate possible regulatory alterations affecting OXPHOS during carcinogenesis, we estimated apparent affinity mitochondria for ADP. In all CRC and polyp groups, the corresponding K_m_(ADP) value was determined and the measured values (Figure 1B, Tables S1 and S2) were found to be 4 to 8 times higher than in isolated mitochondria (15 μM, measured by Chance and Williams [49,50]). This finding points to the existence of restrictions for the movement of ADP through mitochondrial membranes. The OXPHOS system is located in the inner mitochondrial membrane and the ADP/ATP carrier has the function of crossing the adenine nucleotides through the membrane into the mitochondrial matrix. In our previous study, we applied metabolic control analysis on ATP-synthasome which consisted of the respiratory system, ATP-synthase, ATP/ADP carrier and Pi transporter, all in CRC tissue. In the framework of metabolic control analysis and by using specific inhibitors, the rate of effect each enzyme has in a pathway (flux control coefficients) can be determined. This analysis showed that the main control over ATP-synthesis by OXPHOS (the highest flux control coefficients) in CRC relied on respiratory complexes I and III and Pi transporter. Inhibition of the ADP/ATP carrier had no major rate-limiting effect on ATP synthesis by OXPHOS [26]. Thus, we assumed that the considerable control over ability of exogenous ADP to influence respiration was mainly dependent on ADP passage through MOM in CRC. The comparison of K_m_(ADP) values for *KRAS* mutated, *BRAF* mutated and wild-type tumors did not reveal any substantial differences. In all CRC groups the Km(ADP) values for tumor and control tissue were similar. Our previous study showed that we can distinguish two different populations of mitochondria in control tissue—what we believe could be a mucosal population with lower K_m_(ADP) (75 ± 4 μM), and the smooth muscle population with a much higher K_m_(ADP) value (362 ± 60 μM) [25]. This is in good agreement with our preliminary results obtained from separately measured colon smooth muscle and mucosa (259 ± 35 μM and 118 ± 11 μM, respectively). To estimate the percentage of mitochondria with highly regulated (oxidative) and unregulated (glycolytic) MOM permeability, we applied the mathematical model used for muscle cells and adapted it to tissues studied by us. According to the model proposed earlier [51], the hypothetical percentage of low oxidative capacity mitochondria in tissue is calculated from the K_m_(ADP) value as an inverse asymptotic dependence. Percent of low oxidative capacity of mitochondrion demonstrates the metabolic shift to glycolytic state in all colon polyps, but not in *KRAS* mutated and wild-type tumors compared to control tissue *(*Table 1, Tables S1 and S2). The changes in glycolytic markers have been observed in the early premalignant colorectal mucosal field and these changes would be expected to promote increased glycolysis [19]. The K_m_(ADP) values in polyp molecular groups were 55.3 ± 7.4 µM, 52.5 ± 4.7 µM and 60.1 ± 6.3 µM for *KRAS* mutated, *BRAF* mutated and wild-type group, respectively. These were lower than in control tissue (Tables S1 and S2), which indicates significant changes in regulation MOM permeability. Interestingly, despite the similar V_max_ values in *KRAS* mutated polyp and CRC groups, the difference in K_m_(ADP) between these groups was significant, *p* = 0.014 (Tables S1, S2 and Figure S1). Our findings of the relatively low K_m_ value for ADP for colorectal polyps suggest an early metabolic reprogramming towards the glycolytic phenotype with functional OXPHOS.

The results of the current study confirm our previous findings, indicating that in cancer tissues, the regulation of MOM permeability to adenine nucleotides is different from that in normal cells [25,28,29]. Proteins that could regulate the VDAC permeability for adenine nucleotides in colonocytes and corresponding cancer cells are still unknown. There are two possible mechanisms proposed for this regulation. According to the first model, cancer cells due to overexpression of mitochondrially-bound hexokinase 2 support high permeability of the VDAC to adenine nucleotides and direct the ATP formed in mitochondria to the glycolytic pathway. As a consequence, the aerobic glycolysis is facilitated and malignant metabolic reprogramming occurs [52,53]. The second model involves the inhibition of VDAC by free tubulin to limit mitochondrial metabolism in cancer cells [30,54]. The possible candidates are βIII–tubulin and γ-tubulin. βIII–tubulin acts as a marker of cancer aggressiveness, and γ-tubulin formed meshwork has been shown to be associated with mitochondrial membranes [29,55,56]. However, the regulation of energy metabolism through control over metabolites and energy fluxes that pass through the MOM is only one aspect of the possible role of VDAC influencing carcinogenesis. VDAC1—the major mitochondrial protein expressed in mammals and functions in metabolism, Ca^2+^ homeostasis, apoptosis and other activities—is regulated via its interaction with many proteins associated with cell survival and cellular death pathways. VDAC1 is overexpressed in many cancers and represents a promising cancer drug target (reviewed in [57,58]). The mechanistic understanding behind the changes in K_m_(ADP) during CRC carcinogenesis observed in the current study and connections with other functions of VDAC require further investigation.

**Figure 2:**
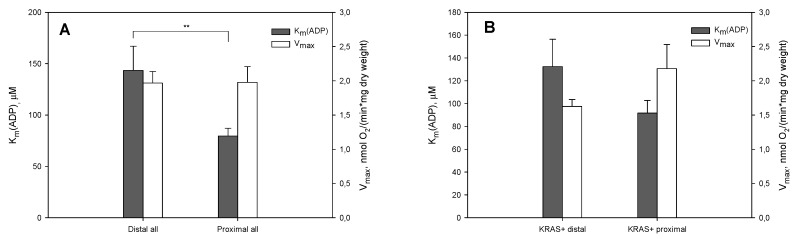
(**A**) In the current study, a comparison of all distal and proximal tumors showed a difference in K_m_(ADP) values, but not in V_max_. (**B**) V_max_ and K_m_(ADP) values comparing proximal and distal tumors in the *KRAS* mutated group. ** Significant difference, *p* < 0.01.

Further, we analyzed whether the observed changes in V_max_ and K_m_(ADP) values are related to tumor location. CRC is more frequently observed in the distal colon (left colon, from splenic flexure to rectum) than in the proximal side (right colon, from the cecum to transverse colon [59]). In the current study, the distal and proximal tumors were presented almost equally—20 and 24 samples, respectively. Studies have shown that tumors arising from the left and right colon are distinct in their epidemiology, biology, histology and microbial diversity [59,60]. In the current study, comparing all the distal and proximal tumors showed differences in K_m_(ADP) but not in V_max_ values (Figure 2A). A study including 57,847 patients showed proximal patients had better outcomes than those with distal CRC in several subgroups including stage II disease, patients aged ˃70 years and mucinous adenocarcinoma [61]. Inside the *KRAS* mutated group, proximal and distal tumors were compared to see the potential effect of cancer location on metabolic changes. No statistically significant difference between V_max_ and K_m_(ADP) values comparing proximal and distal tumors in the *KRAS* mutated group (Figure 2B) was seen. The location of a tumor did not have an effect on the mitochondrial respiration in the *KRAS* mutated group and all observed alterations were related to the *KRAS* status of the tumor. All *BRAF* mutated tumors were located in the proximal side.

All together, we found that colon polyps and colon tumors had higher rates of maximal ADP-activated respiration (a marker of mitochondrial mass) than normal colon tissue (Figure 1A, Tables S1 and S2). *BRAF* mutant tumors and polyps exhibited lower V_max_ values than *KRAS* mutated lesions and they had a relatively high percentage of mitochondria with low control over the movement adenine nucleotides through MOM (Table 1). Therefore, it is most likely that lesions with *BRAF* mutations have higher glycolytic activity, which is confirmed by some published data [62]. In contrast to the *BRAF* mutated lesions, *KRAS* mutated polyps showed signs of stimulated mitochondrial biogenesis and upon progression could give highly metastatic malignant tumors (i.e., polyps with this energetic phenotype can be more prone to tumor formation). This was unexpected, since the transformed cells carrying the *KRAS* gene mutations were characterized by an increased glycolytic flow associated with the over-expression of glucose transporter 1 (GLUT1) and hexokinase 2 and reduced oxygen consumption due to mitochondrial dysfunction in cell cultures [41,63,64]. Our previous studies demonstrated that the oxygen consumption in vitro significantly differed compared to what occurred in vivo [25]. Moreover, the rate of oxidative ATP production of the tumor seems to be a prognostic marker for cancer survival and metastatic potential [22]. The estimation of *KRAS* or *BRAF* mutation status in colorectal pre- and neoplastic lesions could be a predictor of their response to drugs affecting the OXPHOS. Recently, a new class of anticancer drugs called “mitocans” was proposed. These affect different mitochondrial-associated activities including ATP/ADP carrier, hexokinase, electron transport/respiratory chain inhibitors, and others [65].

## 3. Materials and Methods

### 3.1. Reagents

Unless otherwise indicated, all chemicals were purchased from Sigma-Aldrich Chemical Com. (St. Louis, MO, USA) and were used directly without further purification.

### 3.2. Clinical Material

All tumor patients examined (*n* = 33 with ages ranging from 38 to 91 years) had local or locally advanced disease (T2-4 N0-1, M0-1). The patients in the study had not received prior radiation or chemotherapy (Table 2). All subjects gave their informed consent for inclusion before they participated in the study. The study was conducted in accordance with the Declaration of Helsinki, and the protocol was approved by the Medical Research Ethics Committee (National Institute for Health Development, Tallinn, Estonia) of nr.1728.

CRC post operational and normal tissue samples (0.1–0.5 g) were provided by the Oncology and Hematologic Clinic at the North Estonia Medical Centre (NEMC, Tallinn, Estonia). Pathology reports were obtained by the NEMC for each tissue sample. Only primary tumor samples were examined. All investigations were approved by the Medical Research Ethics Committee (National Institute for Health Development, Tallinn, Estonia) and were in accordance with Helsinki Declaration and Convention of the Council of Europe on Human Rights and Biomedicine.

Normal tissue samples were taken from the same location at sites distant from the tumor and they were evaluated for presence of malignant cells. The adjacent control tissues consisted of colonocytes and smooth muscle cells.

Patients with colorectal polyps (*n* = 15) (Table 2) were consecutive patients undergoing a colonoscopy for resection of the polyps at the West Tallinn Central Hospital. After removal, tissue samples were immediately placed in medium B, which consisted of the following: 0.5 mM EGTA, 3 mM MgCl_2_, 60 mM K-lactobionate, 20 mM taurine, 3 mM KH_2_PO_4_, 110 mM sucrose, 0.5 mM dithiothreitol, 20 mM HEPES, 5 µM leupeptin, 2 mg/mL fatty acids free bovine serum albumin (BSA), pH 7.1. All polyps were analyzed immediately after the colonoscopy with quick cancer tests. Only part of the cancer negative polyps was subjected to further analysis for OXPHOS. Due to the limited amount of fresh tissue, *KRAS* and *BRAF* mutation analyses were performed using Formalin-Fixed Paraffin-Embedded (FFPE) samples.

### 3.3. Preparation of Skinned Tumor Fibers and Permeabilization Procedure

Immediately after the surgery, the tissue samples were placed into pre-cooled (4 °C) medium A, which consisted of 20 mM imidazole, 3 mM KH_2_PO_4_, 0.5 mM dithiothreitol, 20 mM taurine, 4 mM MgCl_2_, 100 mM 2-morpholinoethanesulfonic acid, 2.74 mM K_2_Ca-EGTA, 4.72 mM K_2_-EGTA, 5 µM leupeptin and 2 mg/mL BSA [39]. The samples were dissected into small fiber bundles (10–20 mg) and permeabilized in the same medium with 50 μg/mL of saponin. They were mildly stirred for 30 min at 4 °C [39,66]. The obtained permeabilized (skinned) fibers were then washed three times for 5 min in pre-cooled medium B (without leupeptin). After that, samples were kept in medium B at 4 °C until use. The typical dimension of skinned fibers was about 2 × 2 × 2 mm, and one of these pieces was used in oxygraphic experiments.

### 3.4. Oxygraphic Measurements

**Figure 3:**
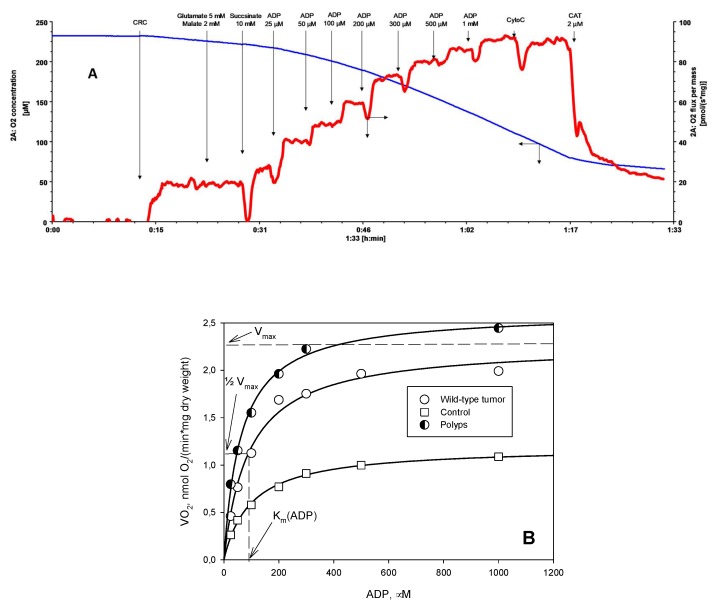
Different kinetics of regulation of mitochondrial respiration by exogenous ADP in colon tissue. (**A**) Recording of original traces of O_2_ consumption by permeabilized colorectal cancer (CRC) tissue upon additions of increasing concentrations of ADP. CAT stands for carboxyatractyloside; CyoC stands for cytochrome C. **(B)** The measured respiration rates were plotted vs ADP concentrations, and from this plot corresponding V_max_ and K_m_(ADP) values were calculated by nonlinear regression using Michaelis–Menten equation. There was a marked difference in ADP kinetics between wild-type CRC, colon polyps and normal colon tissue (control).

Mitochondrial respiration of permeabilized tissue samples was measured at 25 °C in medium B supplemented with 5 mM glutamate, 2 mM malate and 10 mM succinate, with respiratory substrates using a high-resolution respirometer Oxygraph-2k (Oroboros Instruments, Innsbruck, Austria) as described previously [66,67]. The solubility of oxygen at 25 °C was taken as 240 nmol/mL [68]. All respiration rates were normalized per mg dry weight of tissue. To determine the apparent affinity of mitochondria to exogenous ADP (K_m_(ADP)), the dependence of respiration rate on exogenous ADP was measured (Figure 3A). The obtained data were plotted as rates of O_2_ consumption (the basal respiration rate of respiration was subtracted) versus ADP concentration and K_m_(ADP) and V_max_ values were calculated from these plots by nonlinear regression using Michaelis–Menten equation [69,70] (Figure 3B). Additionally, plotting the data to double reciprocal plot gives information about presence of different mitochondrial populations with differently regulated MOM.

### 3.5. DNA Extraction

DNA from formalin-fixed paraffin-embedded tissue (FFPE) samples was extracted using ZYMO Quick-DNA^TM^ FFPE Kit (Zymo Research, Irvine, CA, USA) according to the manufacturer’s instructions. DNA concentrations and quality were measured using the NanoDrop 2000 spectrophotometer (Thermo Scientific, Waltham, MA, USA).

### 3.6. KRAS and BRAF Mutation Analysis

Mutations in *BRAF* codon 600 of exon 15 (V600E) and *KRAS* codon 12 and 13 of exon 2 were screened using High-Resolution Melt (HRM) analysis. Briefly, a 10 µl reaction mix contained 1x HOT FIREPol^®^ EvaGreen^®^ HRM Mix (Solis BioDyne, Estonia), 250 nM of sense and antisense primers (*KRAS*-antisense, 5′- AAATGACTGAATATAAACTTGTGGTAGT-3′; *KRAS*-sense, 5′- TGAATTAGCTGTATCGTCAAGGCACT-3′; *BRAF*-antisense wild-type, 5′-cgccgcgcgccAAAATAGGTGATTTTGGTCT-3′; *BRAF*-antisense mutation, 5′-TAAAAATAGGTGATTTTGGTCTAGCTACA-3′; *BRAF*-sense, 5′- CCACAAAATGGATCCAGACAACTG 3′) and 100× dilution of PCR amplification product. PCR amplification and HRM analysis were performed with Rotor-Gene 6000 (QIAGEN) and consisted of an initial 15 min denaturation step at 95 °C, followed by 45 cycles at 95 °C for 10 s, 54 °C for 10 s and 72 °C for 15 s, with a final extension at 72 °C for 3 min. The resulting PCR products were heated at 95 °C for 1 min and cooled to 40 °C to facilitate heteroduplex formation. HRM analysis was performed from 62 °C to 92 °C with a 0.1 °C step. The results were analyzed using Rotor-Gene 6000 software and unknown samples were compared to control samples with known genotypes.

### 3.7. Data Analysis

Data in the text, tables and figures are presented as mean ± standard error (SEM). Results were analyzed by Student’s *t*-test and *p*-values < 0.05 were considered statistically significant. Apparent K_m_ values for ADP were measured by fitting experimental data to a non-linear regression (according to a Michaelis–Menten model equation, as shown in Figure 3).

## 4. Conclusions

While many studies have characterized the metabolic phenotype of CRC cell lines, it is important to understand the metabolic reprogramming in clinical material. Our findings confirm that early changes in mitochondria respiration occur in CRC carcinogenesis and precede the development of pre-cancerous lesions. Mitochondrial respiration differs in *KRAS*, *BRAF* mutated and wild-type tumor groups, confirming that oncogenes may affect the metabolic requirements of cancer cells. In common polyps, it still remains unclear whether the specific metabolic requirement of tumor cells is dictated by oncogenes or if they change dynamically during tumor evolution. Mitochondrial biogenesis, involved in mitochondrial respiration rate, may be developed to be the prognostic marker for cancer prognosis. As there are profound differences in mitochondrial respiration, the assessment of the metabolic profile of CRC polyps and tumors has the potential to become a component of patient risk stratification.

## Figures and Tables

**Table 1:** Modelled percentage of low oxidative capacity of mitochondrion in KRAS+, BRAF+ and wild-type tumors and controls.

Sample	% of Low Oxidative Capacity of Mitochondrion
*KRAS* tumors	28.1
*KRAS* polyps	65.9
*BRAF* tumors	43.0
*BRAF* polyps	68.6
Wild-type tumors	32.4
Wild-type polyps	61.7
All controls	29.0

**Table 2:** Clinicopathological patient characteristics of the colon cancer and polyps cohort.

Characteristics	*n*
Total patients	48
Females	19
Males	29
Age at diagnosis	
Mean	72
Median	74
Range	38–91
Stage of tumor	
I-II	15
III-IV	9
Unknown	9
Molecular subtype of tumor	
*KRAS* mutated	13
*BRAF* mutated	6
*KRAS* and *BRAF* wild-type	14
Molecular subtypes of polyps	
*KRAS* mutated	4
*BRAF* mutated	2
*KRAS* and *BRAF* wild-type	9

## Supplementary Materials

The following are available online at https://www.mdpi.com/2072-6694/12/4/815/s1, Figure S1: Regulation of mitochondrial respiration in KRAS+, BRAF+ and wild-type tumors and controls, Table S1: The maximal ADP-activated respiration rates (Vmax) comparison by molecular groups. Respiration rates are given in nmol O2/(min×mg dry weight), Table S2: Km comparison by molecular groups.

## Abbreviations

ADP	adenosine diphosphate
CMS	consensus molecular subtype
CRC	colorectal cancer
K_m_	Michaelis–Menten constant
K_m_(ADP)	apparent affinity of mitochondria for exogenous ADP
OXPHOS	oxidative phosphorylation
MOM	outer mitochondrial membrane
VDAC	voltage-dependent anion channel
V_max_	maximal-ADP-activated respiration rate
