# Supplementary material for: Mitochondrial Respiration in KRAS and BRAF Mutated Colorectal Tumors and Polyps
Source: Cancers (Basel). 2020 Mar 28;12(4):815. doi: 10.3390/cancers12040815 (PMC7226330; doi:10.3390/cancers12040815)
Supplement: Supplementary file 1 [file cancers-12-00815-s001.pdf]

Supplementary Materials: Mitochondrial Respiration in *KRAS* and *BRAF* Mutated Colorectal Tumors and Polyps

Egle Rebane-Klemm, Laura Truu, Leenu Reinsalu, Marju Puurand, Igor Shevchuk, Vladimir Chekulayev, Natalja Timohhina, Kersti Tepp, Jelena Bogovskaja, Vladimir Afanasjev, Külliki Suurmaa, Vahur Valvere and Tuuli Kaambre

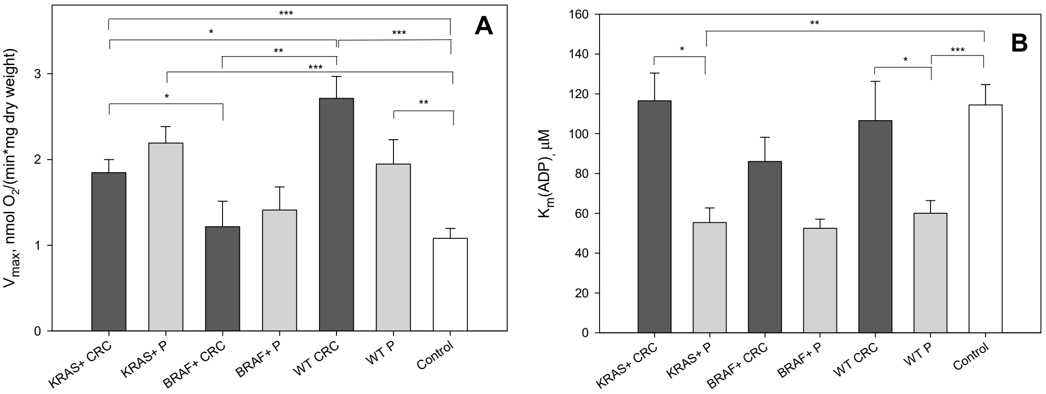

Figure S1. Regulation of mitochondrial respiration in KRAS+, BRAF+ and wild-type tumors and controls. (A) Comparative analysis of maximal respiratory rate (V_{max}) and (B) the apparent Michaelis-Menten constant (K_m(ADP)) values for ADP. KRAS+—*KRAS* mutated; BRAF+—*BRAF* mutated; WT—wild type; CRC—colorectal cancer; P—polyps; Control—control tissue. * $p < 0.05$; ** $p < 0.01$; *** $p < 0.001$.

Table S1. The maximal ADP-activated respiration rates (V_{max}) comparison by molecular groups. Respiration rates are given in nmol O2/(min*mg dry weight) *KRAS*+—*KRAS* mutated; *BRAF*+—*BRAF* mutated; WT—wild-type; CRC—colorectal cancer; P—polyps; Control—control tissue.

**V_{max} Comparison by Molecular Groups**				
**Group (*n*)**	**V_{max} value, nmol O2/(min×mg dry weight)**	**Group (n)**	**V_{max} value, nmol O2/(min×mg dry weight)**	***p* value**
KRAS+ CRC (*n* = 19)	1.93 ± 0.16	BRAF+ CRC (*n* = 7)	1.22 ± 0.30	0.037
KRAS+ CRC (*n* = 19)	1.93 ± 0.16	WT CRC (*n* = 16)	2.67 ± 0.27	0.021
BRAF+ CRC (*n* = 7)	1.22 ± 0.30	WT CRC (*n* = 16)	2.67 ± 0.27	0.004
KRAS+ CRC (*n* = 19)	1.93 ± 0.16	KRAS+ P (*n* = 7)	2.19 ± 0.19	0.383
BRAF+ CRC (*n* = 7)	1.22 ± 0.30	BRAF+ P (*n* = 2)	1.41 ± 0.27	0.755
WT CRC (*n* = 16)	2.67 ± 0.27	WT P (*n* = 13)	1.95 ± 0.28	0.079
KRAS+ CRC (*n* = 19)	1.93 ± 0.16	Control (*n* = 18)	1.08 ± 0.12	<0.001
BRAF+ CRC (*n* = 7)	1.22 ± 0.30	Control (*n* = 18)	1.08 ± 0.12	0.601
WT CRC (*n* = 16)	2.67 ± 0.27	Control (*n* = 18)	1.08 ± 0.12	<0.001
KRAS+ P (*n* = 7)	2.19 ± 0.19	Control (*n* = 18)	1.08 ± 0.12	<0.001
BRAF+ P (*n* = 2)	1.41 ± 0.27	Control (*n* = 18)	1.08 ± 0.12	0.373
WT P (*n* = 13)	1.95 ± 0.28	Control (*n* = 18)	1.08 ± 0.12	0.004

Table S2. K_m comparison by molecular groups. *KRAS*+—*KRAS* mutated; *BRAF*+—*BRAF* mutated; WT—wild type; CRC—colorectal cancer; P—polyps; Control—control tissue.

**K_m Comparison by Molecular Groups**				
**Group (*n*)**	**K_m value, (μM)**	**Group (*n*)**	**K_m value, (μM)**	***p* value**
KRAS+ CRC (*n* = 18)	116.60 ± 13.86	BRAF+ CRC (*n* = 7)	86.02 ± 12.15	0.209
KRAS+ CRC (*n* = 18)	116.60 ± 13.86	WT CRC (*n* = 14)	106.55 ± 19.72	0.671
BRAF+ CRC (*n* = 7)	86.02 ± 12.15	WT CRC (*n* = 14)	106.55 ± 19.72	0.494
KRAS+ CRC (*n* = 18)	116.60 ± 13.86	KRAS+ P (*n* = 7)	55.33 ± 7.40	0.014
BRAF+ CRC (*n* = 7)	86.02 ± 12.15	BRAF+ P (*n* = 2)	52.45 ± 4.67	0.204
WT CRC (*n* = 14)	106.55 ± 19.72	WT P (*n* = 13)	60.07 ± 6.32	0.039
KRAS+ CRC (*n* = 18)	116.60 ± 13.86	Control (*n* = 18)	114.43 ± 10.23	0.901
BRAF+ CRC (*n* = 7)	86.02 ± 12.15	Control (*n* = 18)	114.43 ± 10.23	0.131
WT CRC (*n* = 14)	106.55 ± 19.72	Control (*n* = 18)	114.43 ± 10.23	0.708
KRAS+ P (*n* = 7)	55.33 ± 7.40	Control (*n* = 18)	114.43 ± 10.23	0.002
BRAF+ P (*n* = 2)	52.45 ± 4.67	Control (*n* = 18)	114.43 ± 10.23	0.064
WT P (*n* = 13)	60.07 ± 6.32	Control (*n* = 18)	114.43 ± 10.23	<0.001

© 2020 by the authors. Licensee MDPI, Basel, Switzerland. This article is an open access article distributed under the terms and conditions of the Creative Commons Attribution (CC BY) license (http://creativecommons.org/licenses/by/4.0/).
